# Dynamic changes of angiopoietins and endothelial nitric oxide supply during fluid resuscitation for major gyn-oncological surgery: a prospective observation

**DOI:** 10.1186/s12967-020-02236-9

**Published:** 2020-01-31

**Authors:** Jennifer Gehlen, Sven Klaschik, Claudia Neumann, Mignon-Denise Keyver-Paik, Alexander Mustea, Martin Soehle, Stilla Frede, Markus Velten, Andreas Hoeft, Tobias Hilbert

**Affiliations:** 1 Department of Anesthesiology and Intensive Care Medicine, University Hospital Bonn, Venusberg-Campus 1, 53127 Bonn, Germany; 2 Department of Gynecology and Obstetrics, University Hospital Bonn, Venusberg-Campus 1, 53127 Bonn, Germany

**Keywords:** Angiopoietins, Nitric oxide, eNOS, Microvascular reactivity, Fluid resuscitation

## Abstract

**Background:**

Despite goal-directed hemodynamic therapy, vascular function may deteriorate during surgery for advanced abdominal tumor masses. Fluid administration has been shown to be associated with distinct changes in serum levels of functional proteins. We sought to determine how serum total protein and angiopoietin (ANG) levels change during major abdominal tumor surgery. In addition, ex vivo endothelial nitric oxide synthase (eNOS) activation as well as NO bioavailability in vivo were assessed.

**Methods:**

30 patients scheduled for laparotomy for late-stage ovarian or uterine cancer were prospectively included. Advanced hemodynamic monitoring as well as protocol-driven goal-directed fluid optimization were performed. Total serum protein, ANG-1, -2, and soluble TIE2 were determined pre-, intra-, and postoperatively. Phosphorylation of eNOS was assessed in microvascular endothelial cells after incubation with patient serum, and microvascular reactivity was determined in vivo by near-infrared spectroscopy and arterial vascular occlusion.

**Results:**

Cardiac output as well as preload gradually decreased during surgery and were associated with a median total fluid intake of 12.8 (9.7–15.4) mL/kg*h and a postoperative fluid balance of 6710 (4113–9271) mL. Total serum protein decreased significantly from baseline (66.5 (56.4–73.3) mg/mL) by almost half intraoperatively (42.7 (36.8–51.5) mg/mL, p < 0.0001) and remained at low level. While ANG-1 showed no significant dilutional change (baseline: 12.7 (11.9–13.9) ng/mL, postop.: 11.6 (10.8 –13.5) ng/mL, p = 0.06), serum levels of ANG-2 were even increased postoperatively (baseline: 2.2 (1.6–2.6) ng/mL vs. postop.: 3.4 (2.3–3.8) ng/mL, p < 0.0001), resulting in a significant shift in ANG-2 to ANG-1 ratio. Ex vivo phosphorylation of eNOS was decreased depending on increased ANG-2 levels and ANG-2/1 ratio (Spearman r = − 0.37, p = 0.007). In vivo, increased ANG-2 levels were associated with impaired capillary recruitment and NO bioavailability (Spearman r = − 0.83, p = 0.01).

**Conclusions:**

Fluid resuscitation-associated changes in serum vascular mediator profile during abdominal tumor surgery were accompanied by impaired eNOS activity ex vivo as well as reduced NO bioavailability in vivo. Our results may explain disturbed microvascular function in major surgery despite goal-directed hemodynamic optimization.

## Background

Surgical interventions for abdominal tumor mass reduction are among the most invasive operations in gynecology. Extensive trauma due to deperitonealization together with hemorrhage from rich vascularized tissues result in severe intra- and perioperative volume shifts [1]. These may necessitate large amounts of fluids to be administered to stabilize hemodynamics. Extensive fluid resuscitation in peritoneal cancer patients is associated with a poor postoperative outcome independent from the underlying malignant disease [2, 3]. Therefore, goal-directed fluid therapy (GDT) is recommended to avoid volume overload [4]. However, microvascular function has been shown to deteriorate during major gyn-oncological abdominal surgery despite the use of GDT [5]. Impaired vascular function, in turn, is one determinant of poor outcome after surgery [6, 7].

On one hand, surgery itself induces a SIRS (systemic inflammatory reaction syndrome)-like phenotype. Recently, we could demonstrate that a self-regulating network of mediators for inflammation is highly dysbalanced following cytoreductive surgery for late-stage ovarian cancer [8]. This may promote intra- and postoperative vasodilation and vascular leakage. On the other hand, shifts in plasma and serum levels of functional proteins caused by large-volume fluid resuscitation have a significant impact on clinical endpoints, e.g., dilutional coagulopathy or immune dysfunction [9]. Angiopoietins are key peptides of vascular signaling. Their significance for blood vessel and endothelial dysfunction during systemic inflammation and sepsis is more than well documented [10, 11]. Therefore, it was our aim to assess if their serum levels are altered during fluid resuscitation in major gynecological abdominal tumor surgery and if this is associated with impaired vascular function. We followed a translational approach, comprising clinical data together with analyses of serum samples at various time points and ex and in vivo-assaying, to gain a comprehensive insight.

## Materials and methods

### Study design, patient information

This observational study was conducted in accordance with the declaration of Helsinki and after approval by the institutional review board (IRB) of the University of Bonn (Protocol Number 360/16, date of approval March 21, 2018). Patients being scheduled for laparotomy for advanced-stage ovarian or uterine cancer were prospectively screened to participate in the study. Exclusion criteria were as follows: inability or refusal to provide written informed consent, patient age < 18 years, and pregnancy. Prior to anesthesia induction, a thoracic epidural catheter was usually placed for postoperative analgesia. All patients received anesthesia induction according to standard procedures including intubation, femoral arterial line, central venous catheterization, and urinary catheter. Anesthesia was induced with sufentanil, propofol, and rocuronium and was maintained with either isoflurane or sevoflurane and by continuous infusion of remifentanil. In addition to standard monitoring, advanced hemodynamic monitoring was performed using the VolumeView™ system (EV-1000, Edwards Lifesciences Corp., Irvine, CA, USA). Cardiac index (CI), stroke volume index (SVI), stroke volume variation (SVV), and systemic vascular resistance index (SVRI) were measured continuously by pulse contour analysis, while global enddiastolic volume index (GEDI), intrathoracic blood volume index (ITBI), and extravascular lung water index (ELWI) were assessed discontinuously upon transpulmonary thermodilution. Baseline values were determined before beginning of surgery, and intraoperative values were normalized to the baseline.

Intraoperative hemodynamic management followed a goal-directed algorithm. Crystalloid and colloid fluids were administered to keep CI > 3.0 l/min*m^2^, SVI > 40 mL/m^2^, and SVV < 15%. When CI and SVI were still below target values despite prior fluid optimization, dobutamine was administered additionally. Norepinephrine was administered in case of a mean arterial pressure (MAP) still < 65 mmHg despite prior fluid optimization. Red blood cell and platelet concentrates and Fresh Frozen Plasma were substituted according to recent transfusion guidelines. If intraoperative determination of serum albumin revealed critically low values (< 20 g/l), human albumin solution was substituted on the responsibility of the attending anesthetist that was not part of the study team. Patients that were administered albumin during surgery were excluded from subsequent analysis of total serum protein.

Depending on the progress of cancer, some patients were treated with hyperthermic intraperitoneal chemotherapy. Upon completion of the surgical procedure, anesthesia was terminated, and patients were extubated if a stable respiratory situation was provided. Subsequently, patients were transferred to the ICU for postoperative care.

### Assessment of total serum protein and angiopoietin and soluble TIE2 (sTIE2) levels

Ten mls of blood were drawn before the beginning of surgery (at baseline), intraoperatively every 5000 mL of administered (crystalloid and colloid) infusion solution, and postoperatively. Coagulated samples were centrifuged (3000 rpm, 4 °C, 10 min), and serum aliquots were stored at − 80 °C for subsequent analysis.

Total protein levels were assessed in serum samples using the Pierce™ bicinchoninic acid (BCA) assay kit (Thermo Fisher Scientific, Waltham, MA, USA) according to the manufacturer’s instructions and as described in [12]. Angiopoietin 1 (ANG-1) was measured using a commercially available ELISA kit (RnD Systems, Minneapolis, MN, USA). ANG-2 and sTIE2 levels were detected using custom-made Luminex™ multiplex arrays purchased from RnD Systems according to the manufacturer’s protocol. Bead-based multiplex arrays such as the Luminex™ system are described in [13]. Arrays were analyzed on a MAGPIX™ reader (Luminex Corp., Austin, TX, USA). Results are given in ng/mL serum. All experimental analyses were performed in duplicates. Out of these results, the mean value was calculated and used for further statistical analyses. All personnel performing the serum analyses were blinded for the intra- and postoperative patient data.

### Assessment of endothelial nitric oxide synthase (eNOS) phosphorylation in human dermal microvascular endothelial cells (hdMVEC)

Phosphorylation of eNOS on site serine 1176 (Ser1176) was assessed in hdMVEC ex vivo following incubation with diluted patient serum using the CytoFluor™ eNOS (Phospho-Ser1176) Fluorometric Cell-Based ELISA Kit (FLUO-CBP1542) from Assay Biotechnology (Fremont, CA, USA) according to the manufacturer’s instructions (Fig. 3a). Phosphorylation of eNOS on Ser1176 has been shown to result in increased NO production at basal levels, therefore maintaining microvascular function [14].

Briefly, cryopreserved pooled hdMVEC and the recommended cell culture media were purchased from PromoCell (Heidelberg, Germany). Cells of less than 6 passages were cultured in medium in T75 flasks until 80–90% confluence in a standard cell culture incubator (37 °C in a 5% CO_2_ humidified atmosphere). Cells were harvested using Accutase™ solution (Sigma-Aldrich, St. Louis, MO, USA) and seeded into 96-well plates coated with Attachment Factor (Thermo Fisher Scientific). After overnight cultivation, the supernatant was replaced by patient serum diluted (10%) in cell culture medium. Serum samples collected from different patients at the various time points (baseline, after 5 L of fluid administration, or postoperatively) were used for assaying. Cells were incubated for 4 h, then the supernatant was removed, and cells were fixed using 4% paraformaldehyde solution (Thermo Fisher Scientific). Subsequently, eNOS as well as phospho-eNOS activity were determined fluorometrically and normalized to glyceraldehyde 3-phosphate dehydrogenase (GAPDH) according to the assay kit protocol.

### Assessment of microvascular reactivity and nitric oxide (NO) bioavailability in vivo

Assessment of microvascular reactivity was performed similar to what was described by Kim et al. [6]. Following induction of anesthesia, a near-infrared spectroscopy (NIRS) sensor (Nonin Medical, Inc., Plymouth, MN, USA) was placed on the patient’s left forearm and connected to a Nonin 7600 4-Channel Regional Oximeter (Nonin Medical, Inc.) to measure muscle tissue oxygen saturation (S_t_O_2_). Dynamic response of microvascular reactivity was assessed by an arterial vascular occlusion test (VOT) performed every 30 min. A blood pressure cuff was placed around the patient’s ipsilateral upper arm (Fig. 4a). For VOT, the cuff was inflated to 50 mmHg above the systolic arterial blood pressure. The pressure was maintained for 4 min, and afterwards, the cuff was rapidly and completely deflated. All data were initially stored in the internal memory of the device and transferred to an external computer for further analysis at the end of surgery. Data were visualized, and the following parameters were derived from the resulting VOT curve (Fig. 4b): baseline S_t_O_2_ before VOT maneuver (PreVOT [%]), slope of tissue desaturation during VOT maneuver (DesatVOT [%/min]), time to recover to baseline S_t_O_2_ following deflation of the cuff (RecovVOT [sec]), and size of reactive hyperemic area following VOT (HyperemicVOT [%*sec]).

### Statistical and bioinformatical analysis

Data were transferred into MS Excel (Microsoft Corp., Redmond, CA, USA). Statistical analysis and visualization were performed using GraphPad PRISM 8 (La Jolla, CA, USA). All data are presented as median values with 25th and 75th percentile. Significance of differences between samples from two different time points was tested using the Wilcoxon signed-rank test. Changes over time in intraoperative assessments of microvascular reactivity were compared with the baseline using the Friedman test in case of complete data sets from all included patients. In case of missing values at various time points, Friedman test is inappropriate and Wilcoxon signed-rank test was used instead. Associations between different parameters were assessed using the Spearman rank correlation coefficient. P values < 0.05 were considered statistically significant.

The datasets generated and analyzed during the current study are available from the corresponding author on reasonable request.

## Results

### Patient population

30 patients were prospectively recruited to participate in the study. While in the majority (26 patients), histopathological analysis revealed advanced-stage ovarian cancer, the four remaining patients were diagnosed with invasive carcinoma of the cervix uteri, with endometrial carcinoma, with uterine leiomyoma, and with ovarian granulosa cell tumor. Median patient age was 70 (56–75) years. Table 1 gives an overview of the basic patients’ characteristics and the procedural details.

**Table 1:** Patient and procedural details

Parameter	Median (25th and 75th percentile)
Patient details:	
n	30
Age (years)	70 (56–75)
Body mass index (kg/m^2^)	27.3 (22.5–31.2)
Diagnosed with ovarian cancer (n [%])	26 (87)
Diagnosed with carcinoma of the cervix uteri (n [%])	1 (3)
Diagnosed with endometrial carcinoma (n [%])	1 (3)
Diagnosed with uterine leiomyoma (n [%])	1 (3)
Diagnosed with ovarian granulosa cell tumor (n [%])	1 (3)
Received neoadjuvant oncostatic chemotherapy (n [%])	11 (37)
Procedural details:	
Duration of surgery (min)	429 (293–542)
Duration of anesthesia (min)	595 (446–689)
Duration of mechanical ventilation (min)	1095 (604–1355)
Postoperative ICU therapy (n [%])	27 (90)
Length of stay in ICU (h)	40 (18–72)
Intraop. fluid intake (mL/kg*h)	12.8 (9.7–15.4)
Postop. fluid balance (mL)	6710 (4113–9271)
Intraop. urine output (mL/h)	114 (78–189)
Numbers of crystalloid infusion solution à 500 mL	14 (10–18)
Numbers of colloid infusion solution à 500 mL	1.5 (0.75–2)
Estimated intraop. blood loss (mL/h)	83.8 (42.6–134)
Patients with packed red blood cell (PRBC) transfusion (n [%])	16 (53)
Of those: numbers of PRBC units transfused	2 (2–4)
Numbers of fresh frozen plasma units transfused	0 (0–4)
Numbers of platelet concentrate units transfused	0 (0–0)
Max. norepinephrine dosage (µg/kg*min)	0.09 (0.06–0.15)
Max. dobutamine dosage (µg/kg*min)	0 (0–0)
Postop. serum lactate (mmol/l)	1.1 (0.92–1.28)
Length of stay in hospital (days)	25 (14–31)
Intrahospital mortality (%)	0

### Intraoperative hemodynamics and fluid balance

**Fig. 1:**
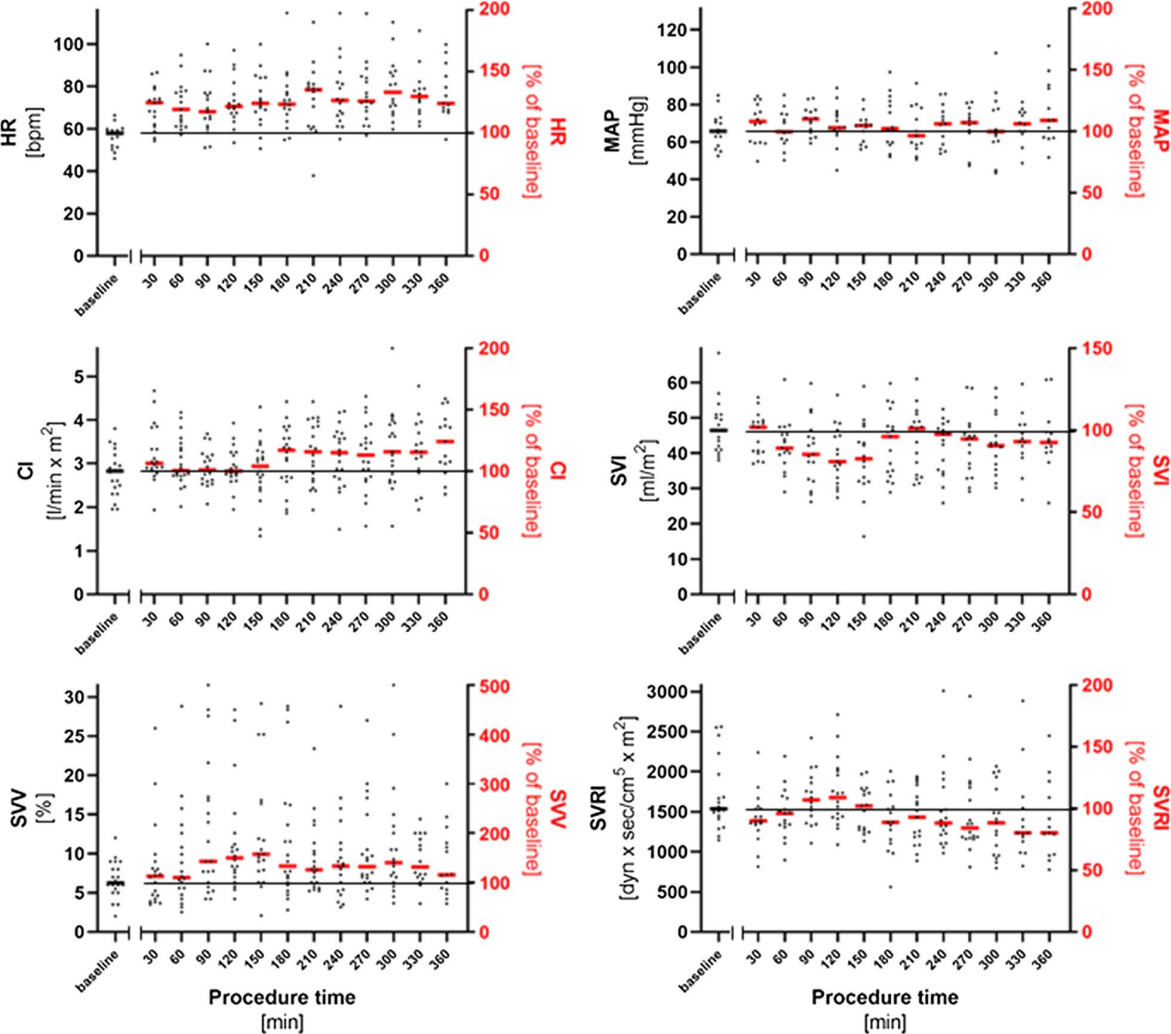
Changes in advanced hemodynamic monitoring during major gynecological abdominal tumor surgery. Advanced hemodynamic monitoring was established after induction of anesthesia and before beginning of surgery. Cardiac index (CI), stroke volume index (SVI), stroke volume variation (SVV), and systemic vascular resistance index (SVRI) were measured continuously by pulse contour analysis. Baseline values before beginning of surgery are plotted on left Y axis (median values indicated by black bar). Intraoperative values were normalized to the baseline and are given as percentage change (plotted on right Y axis, median indicated by red bar) Figure shows absolute values with the respective median, the thin line indicates the level of baseline value (100%). *HR* heart rate, *MAP* mean arterial blood pressure

The median duration of surgery was 429 (293–542) min, while duration of anesthesia was 595 (446–689) min. Continuous data from intraoperative advanced hemodynamic monitoring (VolumeView™ system) were available in 20 patients (Fig. 1). At baseline, median CI was 2.8 (2.3–3.2) l/min*m^2^, SVI was 47 (41–51) mL/m^2^, SVV was 6.3 (5.1–8.8)  %, GEDI was 641 (558–723) mL/m^2^, and SVRI was 1540 (1385–2034) dyn*sec/cm^5^*m^2^. Hemodynamic management followed a goal-directed algorithm, recommending fluid administration to keep cardiac output within an optimal range in case of elevated SVV. While the latter began to gradually increase within the first 2 h of surgery together with a decrease in SVI, crystalloid as well as colloid infusion solution were administered to stabilize stroke volume. This resulted in a median total fluid intake of 12.8 (9.7–15.4) mL/kg*h and a postoperative fluid balance of 6710 (4113–9271) mL. CI tended to increase over the time, while SVRI decreased during the late phase of surgery. The increase in SVV as well as the decrease in SVI were associated with the postoperative fluid balance (SVV: Spearman r = 0.47, p = 0.03; SVI: Spearman r = − 0.63, p = 0.009), resulting in a high postoperative fluid balance in patients that developed a pronounced increase in SVV or decrease in SVI.

### Changes in serum levels of total protein, angiopoietins, and sTIE2 during surgery

**Fig. 2:**
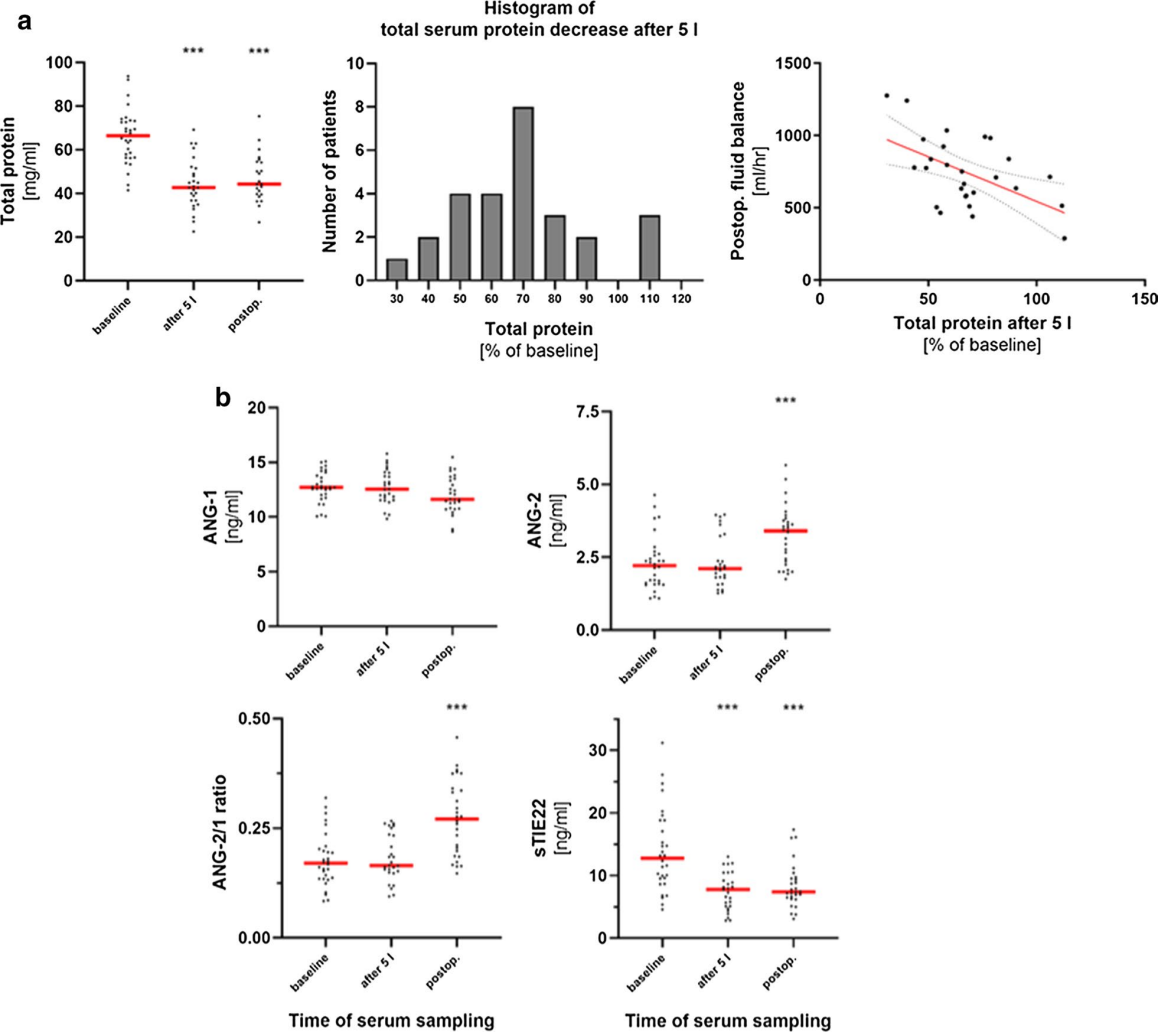
Changes in total serum protein, angiopoietins, and soluble TIE2 during major gynecological abdominal tumor surgery. Serum was sampled before beginning of surgery (baseline), intraoperatively every 5 L of administered fluids, and postoperatively. In samples, total serum protein as well as levels of ANG-1, -2, and soluble TIE2 were determined. Figure shows absolute values for baseline, 5 L, and postoperative time point.** a** Changes in total serum protein are given on the left panel. Middle panel shows the distribution of percentage total serum protein decrease in the whole cohort after 5 L of fluid administration. On the right panel, the association between individual percentage total serum protein decrease after 5 L of fluid administration and the postoperative fluid balance is shown (Spearman rank correlation, dashed lines indicate the 95% confidence interval).** b** Graphs show changes in ANG-1, -2, the ratio between ANG-2 and -1, and sTIE2. Wilcoxon signed-rank test, *** p < 0.001. Changes of serum parameters over time are shown as absolute values with median

According to the study protocol, serum was sampled at baseline, intraoperatively every 5000 mL of administered (crystalloid and colloid) infusion solution, and postoperatively. While serum samples after 5 L of administered fluid were available in all but three patients, only 10 patients received ≥ 10 L, three ≥ 15 L, and only one patient ≥ 20 L. Therefore, Fig. 2a shows the representative changes in serum proteins during the procedure for the baseline, the 5 L, and the postoperative time point. Total serum protein significantly decreased from baseline values of 66.5 (56.4–73.3) mg/mL by factor 0.6 following intraoperative fluid administration to 42.7 (36.8–51.5) mg/mL (p < 0.0001) and remained at a low level. While soluble TIE2 levels were reduced in a similar way, ANG-1 showed no significant change following surgery (baseline: 12.7 (11.9–13.9) ng/mL, postop.: 11.6 (10.8–13.5) ng/mL, p = 0.06) (Fig. 2b). Serum levels of ANG-2 were even significantly increased postoperatively (baseline: 2.2 (1.6–2.6) ng/mL, postop.: 3.4 (2.3–3.8) ng/mL, p < 0.0001), resulting in a likewise significant shift in ANG-2 to -1 ratio by factor 1.6. Of note, there was a wide variation in the extent of total serum protein decrease among the individual patients despite a fixed amount of administered fluids of 5 L at the time of serum sampling (Fig. 2a). The reduction in individual total serum protein was associated with the increase in SVV (Spearman r = − 0.49, p = 0.03) as well as with the postoperative fluid balance (Spearman r = − 0.41, p = 0.03). Moreover, there was a significant association of postoperative individual decrease in ANG-1 serum levels with total fluid intake (Spearman r = − 0.63, p = 0.0004) as well as with postoperative fluid balance (Spearman r = − 0.51, p = 0.005).

### eNOS phosphorylation ex vivo and nitric oxide bioavailability in vivo

**Fig. 3:**
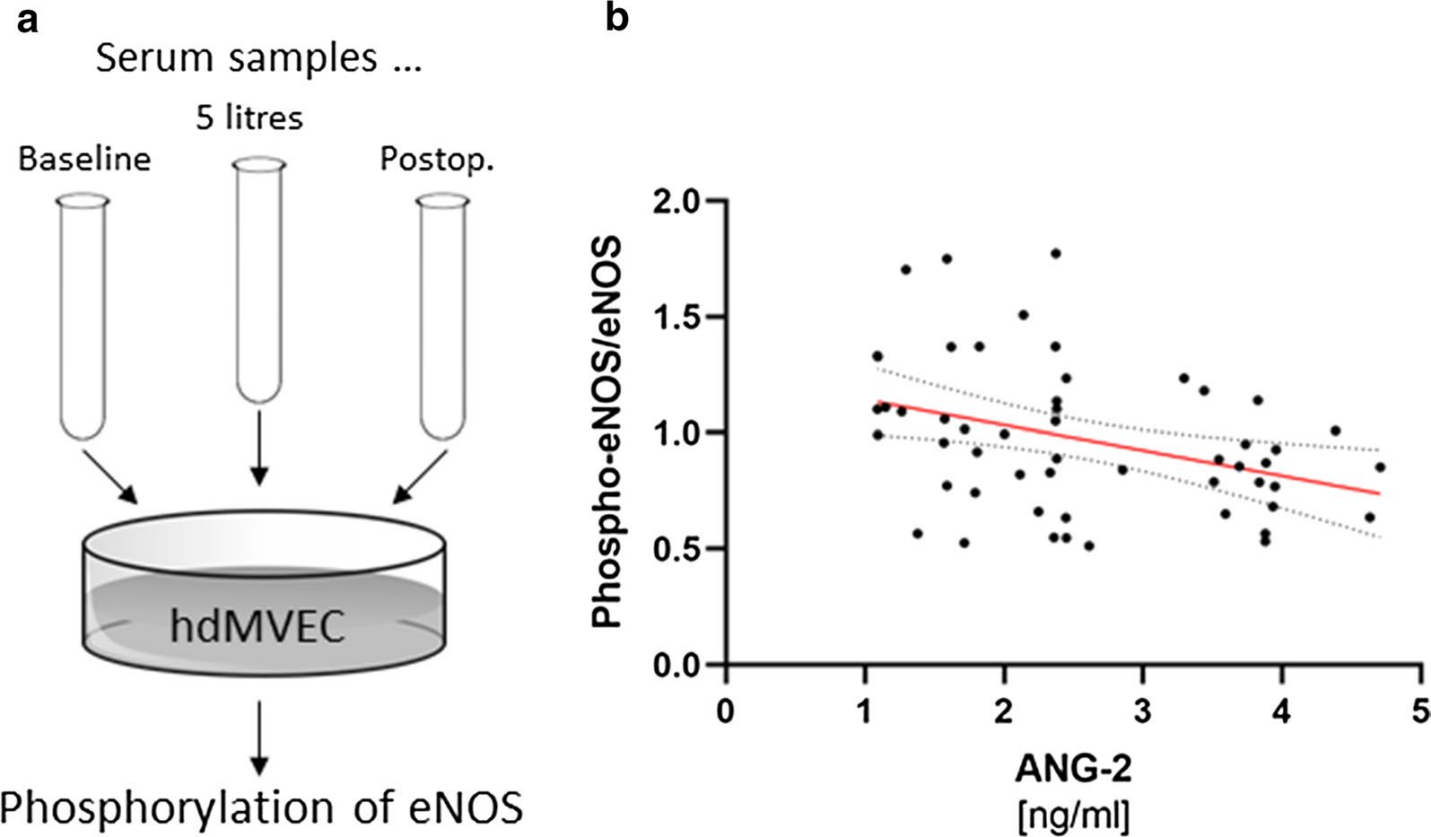
Influence of patient serum on endothelial nitric oxide synthase (eNOS) phosphorylation. ** a** Human dermal microvascular endothelial cells (hdMVEC) were incubated with diluted serum samples collected from different patients at the various time points (baseline, after 5 L of fluid administration, and postoperatively), and eNOS phosphorylation was determined fluorometrically.** b** Graph shows association between individually increased serum ANG-2 levels and a reduction in eNOS phosphorylation ex vivo (Spearman rank correlation, dashed lines indicate the 95% confidence interval)

To assess a possible influence of changing serum mediator levels on vascular cell function, phosphorylation of human dermal microvascular endothelial cell nitric oxide synthase (eNOS) ex vivo as a surrogate for endothelial NO production was measured after incubating cells with diluted serum samples from different patients collected at the various time points (baseline, after 5 L of fluid administration, or postoperatively) (Fig. 3a). As shown in Fig. 3b, increasing ANG-2 content in serum samples was associated with decreased eNOS phosphorylation in hdMVEC (Spearman r = − 0.37, p = 0.007). Of note, the increase in ANG-2/1 ratio was likewise correlated with impaired eNOS phosphorylation (Spearman r = − 0.28, p = 0.04).

**Fig. 4:**
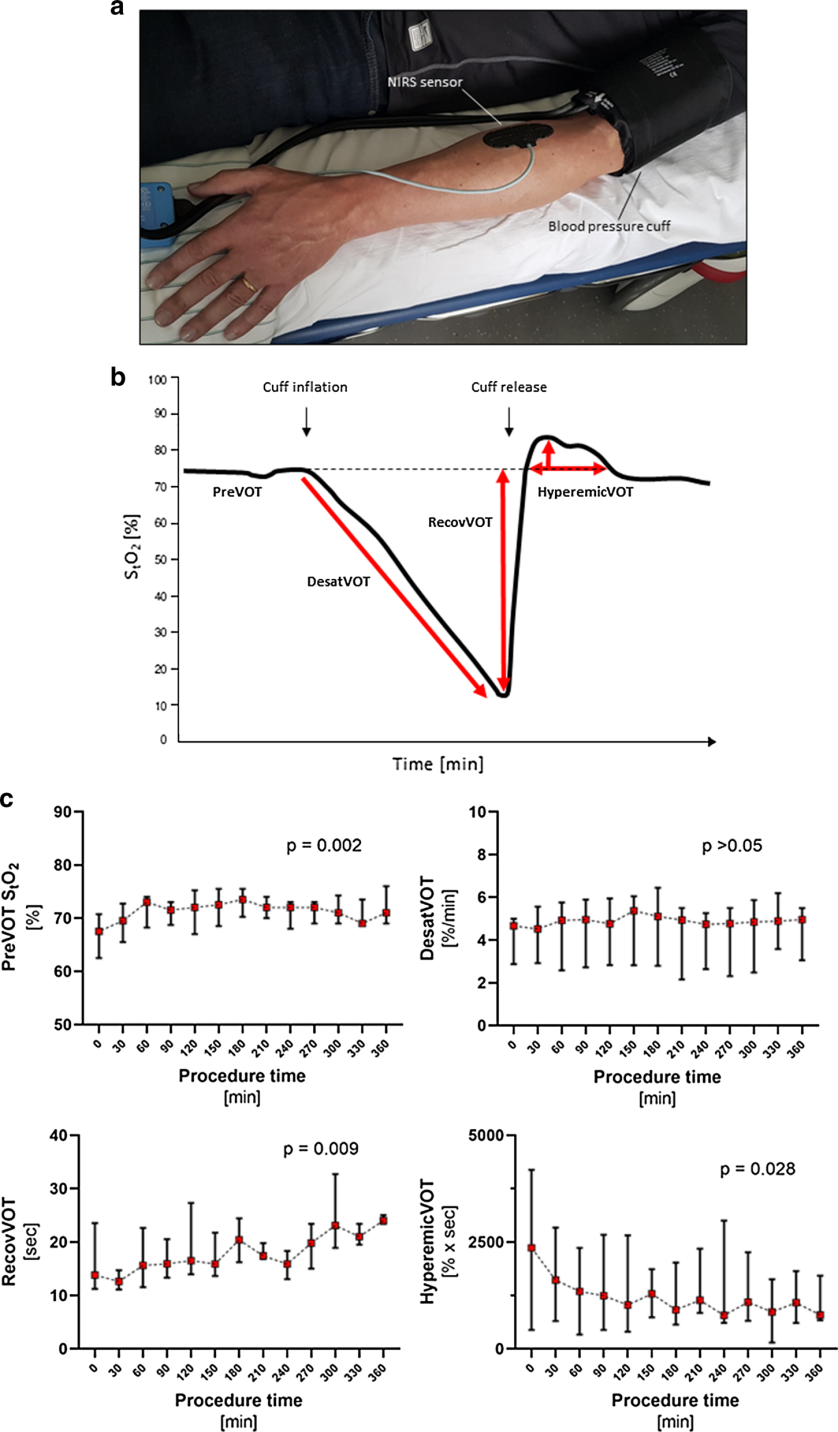
Assessment of dynamic microvascular reactivity and nitric oxide bioavailability during major gynecological abdominal tumor surgery. Dynamic microvascular reactivity was assessed before beginning of and every 30 min during surgery by measuring tissue oxygenation by near-infrared spectroscopy (NIRS) combined with an arterial vascular occlusion maneuver (VOT). ** a** Image illustrates general setup of assessment. A NIRS sensor is placed on the left forearm and connected to the NIRS monitor. A blood pressure cuff is placed around the ipsilateral upper arm. For VOT maneuver, the cuff is inflated to 50 mmHg above the systolic arterial blood pressure, and the pressure is maintained for 4 min before the cuff is rapidly and completely deflated.** b** Muscle tissue oxygen saturation (S_t_O_2_) during VOT maneuver. The following parameters can be derived from the VOT curve: baseline S_t_O_2_ before VOT maneuver (PreVOT), slope of tissue desaturation during VOT maneuver (DesatVOT), time to recover to baseline S_t_O_2_ following deflation of the cuff (RecovVOT), and size of reactive hyperemic area following VOT (HyperemicVOT). ** c** Changes of VOT parameters over time during major gynecological abdominal tumor surgery. Values were compared with the respective baseline using the Friedman test in case of complete data sets from all included patients. In case of missing values at various time points, Friedman test is inappropriate and Wilcoxon signed-rank test was used instead

Dynamic microvascular reactivity was assessed during surgery in a subset of 8 patients by measuring tissue oxygenation by NIRS combined with arterial vascular occlusion (Fig. 4a, b). While general tissue oxygen delivery increased, as evidenced by gradually increasing PreVOT saturation, dynamic muscle desaturation during vascular occlusion was not affected (Fig. 4c). In contrast, parameters indicating microvascular reactivity and capillary recruitment (RecovVOT, HyperemicVOT) were significantly impaired during surgery. Intraoperative individual high serum ANG-2 levels were associated with a decreasing or even abolished post-occlusion hyperemic area (Spearman r = − 0.83, p = 0.01), suggesting reduced endothelial NO bioavailability in these patients. Clinically, fold changes in increasing serum ANG-2 were positively associated with elevated postoperative serum lactate levels as surrogate parameter for impaired microvascular perfusion (Spearman r = 0.56, p = 0.002).

## Discussion

With this study, we sought to assess an impact of fluid resuscitation during major gynecological abdominal tumor surgery on serum levels of angiopoietins. We could show that parameters of advanced hemodynamic monitoring were associated with high intraoperative fluid intake as well as with decreasing total serum protein and altered levels of ANG-1, -2 and soluble TIE2. Moreover, dynamic changes in serum mediator profile were accompanied by impaired phosphorylation of endothelial NOS ex vivo and by progressively disturbed microvascular reactivity and NO bioavailability in vivo. Our data suggest a significant impact of extensive fluid administration on the dynamics of key mediators of vascular function.

Major abdominal surgery can have a pronounced impact on systemic hemodynamics. Maintaining the balance between adequate organ perfusion on one side and hypervolemia on the other often makes intraoperative fluid management challenging. When surgery is performed for advanced stages of solid malignancies such as intestinal or ovarian cancer, excessive release of proinflammatory mediators, severe blood loss, and prolonged operative time may even aggravate hemodynamic deterioration [8, 15]. Recent expert opinion therefore recommends the use of advanced hemodynamic monitoring with dynamic parameters of cardiac preload and output, combined with GDT strategies [4]. In our patients, decreasing SVI together with an increase in SVV were indicative of reduced preload and prompted the anesthetist to administer fluids according to the algorithm. Subsequent normalization of SVI demonstrated the validity this approach for predicting fluid responsiveness in abdominal surgery patients, as already shown by others [16]. Degree of hemodynamic alterations varied among the patients, and as expected, postoperative positive fluid balance was most pronounced in those that developed distinct changes in SVI and SVV.

Marked changes in plasma and serum protein profiles during surgery with an open abdominal cavity may be multicausal. Besides an acute-phase response leading to a strong elevation of proinflammatory mediators [8, 17], increased endothelial permeability provokes leakage not only of albumin but also of functional serum proteins [18, 19]. Hemodilution induced by fluid administration may further alter systemic protein levels. This has been shown to be of substantial clinical impact, e.g. by inducing bleeding due to dilutional coagulopathy and thereby increasing perioperative morbidity [9, 20]. We found an initial rapid decrease in total serum protein to almost half the baseline values during the first period of surgery with a subsequent stabilization at low levels. This is in line with results from other authors [18]. Interestingly, there was a wide variation in total serum protein decrease despite a fixed amount of fluids being administered in every patient at the time of serum sampling. Therefore, dilution may not be the only underlying cause. Other factors, e.g. protein extravasation due to an individual preexisting vulnerability to leakage in the cancer patients, may also play a role in the development of intraoperative hypoalbuminemia. Reports on the impact of localized malignancies on systemic vasculature support this assumption [21]. Decreasing total serum protein was associated with a positive postoperative fluid balance in our patients, suggesting that a reduced oncotic pressure may pose the patient at risk for fluid overload [22].

Although in the whole cohort there was only a trend towards decreasing ANG-1 levels during surgery, postoperatively, they were highly significantly associated with the total fluid intake, and dilution may be a substantial contributing factor. In contrast to ANG-1, ANG-2 levels showed a marked increase. On one hand, this results from the proinflammatory phenotype induced by surgery [8]. On the other hand, it has been demonstrated that the sole administration of large amounts of crystalloid fluids is suffice to raise systemic ANG-2 levels. This is independent of inflammatory activation but probably mediated by changes in endothelial shear stress [23–25]. In sum, in our patients, this resulted in a significant increase in the ANG-2/1 ratio throughout surgery. Such increase in ANG-2/1 ratio is an important risk factor for a worsened perioperative outcome, as we previously demonstrated [10].

Angiopoietin dysbalance towards activating ANG-2 with concurrent reduction of vasoprotective ANG-1 affects tissue and organ perfusion in several ways. The induction of vascular leakage with subsequent development of interstitial edema results in impaired capillary blood flow and an extended diffusion distance for oxygen [10, 26, 27]. Proper function of the endothelial NO synthase is a cornerstone of microcirculation since it mediates capillary autoregulation and recruitment, thereby ensuring homogenous tissue perfusion [28]. Binding of constitutively secreted ANG-1 to its corresponding endothelial receptor TIE2 induces activation of the PI3K/Akt pathway with subsequent phosphorylation of eNOS, which is crucial for vascular integrity [29, 30]. ANG-2 as the natural competitive antagonist for ANG-1 inhibits binding of the latter to TIE2 and thereby abolishes constitutive PI3K/Akt and eNOS activation [31]. Therefore, a disturbed balance between serum ANG-1 and -2 alters eNOS-dependent regulation of perfusion in the microvascular bed [30]. In sepsis, microvascular reactivity, which is directly related to eNOS function, has been shown to be markedly reduced in response to increased systemic ANG-2 levels [32]. When we incubated microvascular endothelial cells with patient serum sampled prior to and during and following surgery, phosphorylation of eNOS on activating site Ser1176 was reduced depending on increasing ANG-2 content of the serum. This suggests impaired NO production mediated by angiopoietin dysbalance.

In addition to sepsis, dynamic microvascular reactivity was demonstrated to deteriorate during major abdominal surgery despite intraoperative goal-directed hemodynamic optimization [5]. We can confirm this. In particular, parameters indicating disturbed capillary recruitment and endothelial NO bioavailability such as the recovery time and the reactive hyperemia following reperfusion were associated with individually increased serum ANG-2 levels in our patients. Impaired microvascular function was shown to be a marker as well as a determinant of a worsened clinical outcome following surgery [6, 7, 33]. In our cohort, increased serum ANG-2 as well as concomitantly reduced microvascular function were associated with postoperatively elevated lactate levels, being indicative of an impaired capillary perfusion during surgery.

Of course, our study has significant limitations. Limited cohort size, especially in the subgroup assessing microvascular reactivity in vivo, is one. Potential heterogeneity in the patient population may produce some bias in our observation, and we did not adjust for this. The selection of mediators and the restriction to angiopoietins possibly limit the validity of the results. However, we focused on ANG-1 and -2 as one of the most significant vascular signaling system due to its importance for the control of capillary autoregulation. The latter has been shown to get lost during major gyn-oncological abdominal surgery previously. Furthermore, our report lacks final causal proofs for the presented associations between fluid resuscitation and impaired NO availability. The results are hypothesis-generating and shed light on the interaction between systemic hemodynamics, circulating and paracrine vascular mediators, and endothelial function in a patient population at risk. They should therefore prompt for further research, also including non-tumor-bearing patients to be compared with (Additional file 1: Fig. S1).

## Conclusions

Our study is the first to demonstrate fluid resuscitation-associated changes in serum vascular and endothelial mediator profile that are in concert with hemodynamic parameters as well as concomitant deterioration of microvascular function during major abdominal surgery. Angiopoietin kinetics were associated with impaired endothelial NOS activity ex vivo and with reduced NO bioavailability in vivo. Our data therefore provide a possible explanation for disturbed microvascular function in major abdominal surgery despite goal-directed hemodynamic optimization. Furthermore, they stress the vulnerability of such patients for fluid overload and its possible adverse effects.

## Supplementary information

**Additional file 1: Figure S1.** Association between systemic hemodynamics, circulating and paracrine vascular mediators, and endothelial and microvascular function ex vivo and in vivo in patients undergoing major gyn-oncological abdominal surgery.

## Data Availability

The datasets used and/or analyzed during the current study are available from the corresponding author on reasonable request.

## Abbreviations

GDT: Goal-directed (fluid) therapy
SIRS: Systemic inflammatory reaction syndrome
IRB: Institutional review board
CI: Cardiac index
SVI: Stroke volume index
SVV: Stroke volume variation
SVRI: Systemic vascular resistance index
GEDI: Global enddiastolic volume index
ITBI: Intrathoracic blood volume index
ELWI: Extravascular lung water index
MAP: Mean arterial blood pressure
HR: Heart rate
sTIE2: Soluble TIE2
BCA assay: Bicinchoninic acid assay
ANG: Angiopoietin
eNOS: Endothelial nitric oxide synthase
hdMVEC: Human dermal microvascular endothelial cells
Ser1176: Phosphorylation site serine 1176
GAPDH: Glyceraldehyde 3-phosphate dehydrogenase
NIRS: Near-infrared spectroscopy
StO2: Muscle tissue oxygen saturation
VOT: Arterial vascular occlusion test
PreVOT: Baseline StO2 before VOT maneuver
DesatVOT: Slope of tissue desaturation during VOT maneuver
RecovVOT: Time to recover to baseline StO_2_ following deflation of the cuff
HyperemicVOT: Size of reactive hyperemic area following VOT
