# Supplementary figures and images for: Dynamic changes of angiopoietins and endothelial nitric oxide supply during fluid resuscitation for major gyn-oncological surgery: a prospective observation
Source: J Transl Med. 2020 Jan 31;18:48. doi: 10.1186/s12967-020-02236-9 (PMC6995240; doi:10.1186/s12967-020-02236-9)

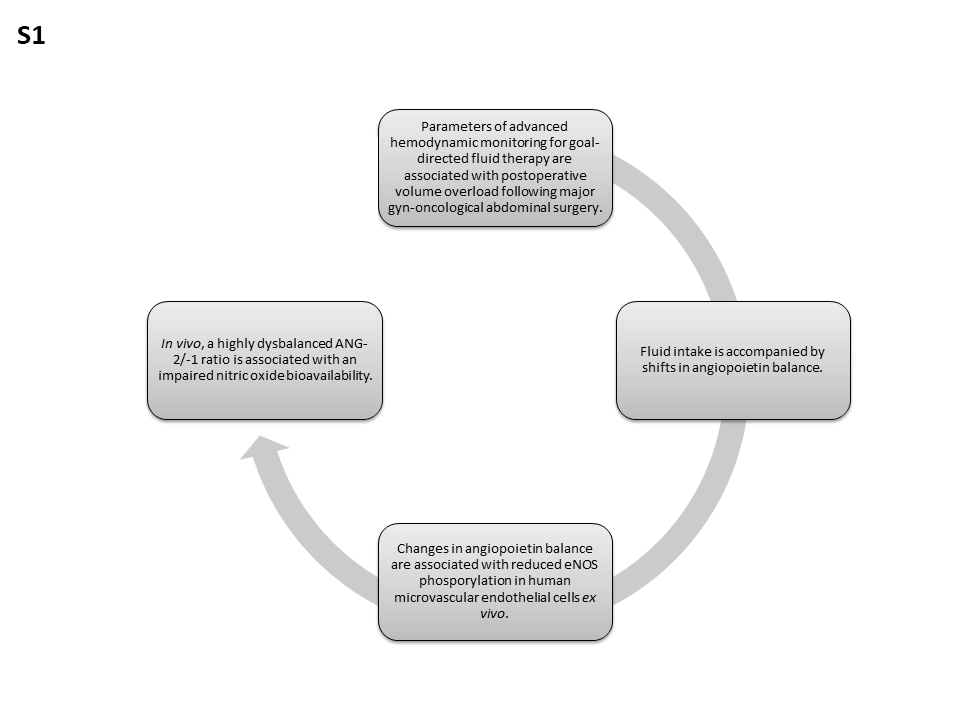

Supplement: Supplementary file 1 — Additional file 1: Figure S1. Association between systemic hemodynamics, circulating and paracrine vascular mediators, and endothelial and microvascular function ex vivo and in vivo in patients undergoing major gyn-oncological abdominal surgery. [file 12967_2020_2236_MOESM1_ESM.tif]
